# Comparative Evaluation of Hyaluronic Acid (hyaDENT BG^®^ Gel) and Enamel Matrix Proteins (Emdogain^®^) in the Regenerative Treatment of Angular Bone Defects Using Xenograft (Bio-Oss Collagen^®^)—A Clinical Trial

**DOI:** 10.3390/jfb16120431

**Published:** 2025-11-24

**Authors:** Velitchka Dosseva-Panova, Hristina Maynalovska, Antoaneta Mlachkova, Ekaterina Tosheva, Ivan Ivanov, Zdravka Pashova-Tasseva

**Affiliations:** 1 Department of Periodontology, Faculty of Dental Medicine, Medical University of Sofia, 1431 Sofia, Bulgaria; v.doseva@fdm.mu-sofia.bg (V.D.-P.); a.mlachkova@fdm.mu-sofia.bg (A.M.); ivan.ivanov@fdm.mu-sofia.bg (I.I.); z.pashova@fdm.mu-sofia.bg (Z.P.-T.); 2 Department of Statistics and Econometrics, Faculty of Applied Informatics and Statistics, University of National and World Economy, 1000 Sofia, Bulgaria; etosheva@unwe.bg

**Keywords:** angular bone defects, enamel matrix derivative, hyaluronic acid, periodontal regenerative therapy, periodontitis

## Abstract

Biomolecules have gained attention in recent years for their potential to enhance regenerative periodontal therapy. This study compared hyaluronic acid (HA) and enamel matrix derivative (EMD), each combined with a xenogeneic graft, in adults with periodontitis presenting vertical bone loss with an intrabony component ≥3 mm. Seventeen participants contributed 28 defects, assigned in equal numbers to Bio-Oss Collagen^®^ plus HA gel (hyaDENT BG^®^) or Bio-Oss Collagen^®^ plus EMD (Emdogain^®^). Outcomes included reduction in probing pocket depth (PPD), gain in clinical attachment level (CAL), and radiographic measures of residual defect and bone fill, assessed at baseline and 6 months after the surgery. Both approaches produced significant within-group improvements in PPD, CAL, and radiographic bone fill (all *p* = 0.001). Postoperative values and mean changes did not differ significantly between groups (all *p* > 0.24). Within the limitations of this small, non-randomized study, the findings indicate that HA gel can achieve clinical and radiographic outcomes comparable to EMD when both are used with a xenogeneic scaffold. These results should be considered preliminary, suggesting that HA may represent a practical and biologically compatible alternative to EMD, particularly in cases where cost, availability, or religious considerations limit its use. Confirmation through larger, randomized, and long-term studies is warranted to validate these observations.

## 1. Introduction

Periodontitis is a widespread inflammatory-destructive disease caused by pathogenic bacterial biofilm, ranking among the six most common diseases in modern society. It can lead to tooth loss and carries significant social and economic implications [1,2,3]. The destruction of tooth-supporting tissues is manifested by clinical attachment loss, radiographically detectable bone loss, periodontal pocket formation, and bleeding on probing. Bone defects associated with periodontal pockets represent a key risk factor for further progression and additional attachment loss if left untreated [4].

In recent years, the concept of tissue-based bioengineering has emerged, grounded in the use of signaling molecules—such as growth factors, scaffolds, and cells—to promote angiogenesis, osteogenesis, and modulation of inflammation. These innovative approaches aim to enhance healing and facilitate periodontal regeneration. Broadly, three types of biomaterials are commonly applied in clinical practice within this framework: bone substitutes, barrier membranes, and signaling molecules (including enamel matrix proteins and others). These materials may be used alone or in various combinations to support regenerative outcomes [5,6,7,8,9].

### 1.1. Bone Substitutes and Bone Filling

Various surgical techniques have been proposed for the treatment of periodontitis and its associated bone defects, with primary goals including the reduction in pocket depth and promotion of attachment gain. The practice of filling bony defects in periodontology has a long history, employing a range of materials such as autogenous bone, demineralized allogenic bone, xenogenic materials, and alloplastic substitutes [10,11]. Ideally, these materials should combine osteoconductive, osteoinductive, and osteogenic properties, although most provide only osteoconduction. Autogenous bone remains the gold standard due to its regenerative potential, but requires a second surgical site. Allografts show variable results depending on donor factors and are restricted in some countries. Xenografts generally offer moderate regeneration, often enhanced by combining them with barrier membranes [12,13,14].

### 1.2. Barrier Membranes

A number of researchers emphasize the importance of early cellular repopulation of the root surface following periodontal surgery in determining whether healing results in repair or true regeneration. Barrier membranes play a crucial role by mechanically excluding the faster-migrating epithelial and connective tissue cells, which compete with the slower-moving osteoprogenitor cells from the periodontal ligament, bone, and cementum. This selective exclusion facilitates an environment more favorable for regeneration [12,15,16,17,18].

### 1.3. Biomolecules

Growth factors and signaling molecules are proteins capable of enhancing chemotaxis, cell proliferation and differentiation, extracellular matrix synthesis, and angiogenesis. Increasing attention has been given in the literature to the osteoinductive potential of growth factors [19,20,21].

Results from numerous preclinical and clinical studies have led to the introduction of various growth factors into the commercial market for the purpose of periodontal regeneration. Among these, enamel matrix derivative (EMD) has become the most widely used and extensively studied agent for promoting periodontal tissue regeneration. Emdogain^®^ (Straumann AG, Basel, Switzerland) is a fully resorbable material composed of EMD extracted from embryonic porcine tooth enamel and suspended in a propylene glycol alginate (PGA) carrier. The active components—enamel matrix proteins—are secreted by Hertwig’s epithelial root sheath and play a pivotal role in odontogenesis, particularly in the formation of cementum, periodontal ligament, and alveolar bone. Controlled clinical studies demonstrate that, for angular defects, open-flap surgery combined with enamel matrix derivatives yields significantly greater change in clinical attachment level (CAL) and radiographic bone fill than open-flap debridement alone. These improvements are accompanied by meaningful reductions in probing pocket depth (PPD). Emdogain^®^ can be used as a stand-alone biologic or in combination with bone substitutes, with the choice guided by defect morphology and soft-tissue conditions [19,20,21,22]. Nevertheless, wider adoption may be tempered by cost considerations, the porcine origin of the extract, and patient preferences—including religious or cultural restrictions.

In recent years, hyaluronic acid (HA) has seen growing clinical adoption as a biologically active adjunct in regenerative periodontal surgery. It is an anionic, non-sulfated glycosaminoglycan and a structured biomolecule that serves as a key component of the extracellular matrix. It is found in nearly all organs and tissues, including the periodontium. In periodontal tissues, HA is synthesized by fibroblasts and keratinocytes in the gingiva, as well as by cells of the periodontal ligament, cementoblasts, and osteoblasts. In dentistry, in vitro and animal studies have shown that hyaluronic acid protects against free oxygen radicals, promotes wound healing, induces angiogenesis, and does not inhibit osteogenesis. Literature reports indicate that HA possesses bacteriostatic, fungistatic, anti-inflammatory, antiedematous, osteoinductive, and proangiogenic properties. Moreover, HA plays a vital role in inflammation, coagulation, granulation, tissue formation, cell migration, and differentiation during regeneration and healing of both soft and hard tissues [23,24,25,26,27,28,29,30,31].

Although both enamel matrix derivatives and hyaluronic acid are widely used in contemporary periodontal practice, direct comparative clinical studies remain limited. The regenerative efficacy of EMD has been extensively documented over the past three decades, whereas evidence for HA in periodontal regeneration is comparatively modest. Given that HA is a natural constituent of periodontal tissues, is biocompatible with blood, and is generally more accessible financially—while EMD is a well-established but costlier porcine-derived product—robust comparative data are needed to determine whether HA can serve as a reliable alternative for regenerative periodontal therapy. In light of this gap, the aim of the present study was to evaluate the efficacy of hyaluronic acid compared with enamel matrix derivatives, each combined with a xenogeneic bone graft, in the treatment of intraosseous periodontal defects.

## 2. Materials and Methods

This was a prospective, non-randomized, two-arm comparative clinical trial. The study was registered on ClinicalTrials.gov under the identifier NCT07230522, with the most recent update posted on 17 November 2025.

### 2.1. Study Population

The study population comprised a convenience sample of patients diagnosed with periodontitis and presenting with vertical bone loss with an infraosseous defect component of ≥3 mm. The number of surgical sites treated was determined by the availability of eligible patients and research funding; no a priori sample size or power calculation was performed. Participants were assigned at the patient level to one of two treatment groups based on case availability, without random allocation.

Group 1 involved regenerative periodontal therapy comprising bone grafting with Bio-Oss Collagen^®^ combined with hyaluronic acid (hyaDENT BG^®^ gel, BioScience GmbH, Dümmer, Germany).

Group 2 was treated with regenerative periodontal therapy involving bone grafting with Bio-Oss Collagen^®^ combined with enamel matrix proteins (Emdogain^®^).

Each group included 14 distinct surgical sites. The distribution of treated sites according to jaw and tooth type is presented in Supplementary Table S1.

The individuals enrolled in the study met predefined eligibility criteria, related either to their general health status or to specific conditions at the periodontal site.

Patient-related inclusion criteria were as follows:

- Adults over the age of 18 diagnosed with periodontal disease—Stage III or IV periodontitis;
- Sites with bone defects and PPD ≥ 6 mm, accompanied by bleeding on probing (BoP) at re-evaluation, conducted six weeks after non-surgical periodontal therapy;
- Full Mouth Plaque Score (FMPS) < 20% and Full Mouth Bleeding Score (FMBS) < 15% prior to surgical treatment;
- Good general health with no systemic conditions and no known allergies to materials or medications used in the study.

Site-specific inclusion criteria included the following:

- Proximal angular bone defects;
- Two-wall or three-wall bone defects;
- At least one defect with PPD ≥ 6 mm, CAL ≥ 5 mm, and an infraosseous component of the defect measuring ≥ 3 mm;
- Vital teeth or teeth with adequately performed endodontic treatment.

Exclusion criteria for the study were as follows:

- Medical conditions contraindicating surgical intervention;
- Pregnancy or lactation;
- Heavy smokers;
- Untreated periodontal disease;
- Poor oral hygiene;
- Acute infectious lesions in the area of intervention;
- Teeth with increased mobility (Grade II or III);
- Restorations or carious lesions on root surfaces associated with the bone defect.

The above criteria were used to minimize surgical risk and to avoid factors known to impair periodontal wound healing (e.g., heavy smoking, poor plaque control, acute infection) or to introduce major confounders (e.g., systemic conditions that influence regenerative outcomes). This approach was intended to strengthen internal validity and participant safety.

### 2.2. Clinical Measurements

The recorded clinical parameters were: oral hygiene index, assessed using the FMPS; gingival status, evaluated using the FMBS; PPD; CAL; BoP; furcation involvement (F); tooth mobility (M); and gingival recession (GR). All measurements were documented in a periodontal chart at baseline, at re-evaluation (6 to 8 weeks after non-surgical periodontal therapy), and at the 6-month postoperative follow-up. Clinical measurements were performed using a UNC-15 periodontal probe.

All assessments were performed by a single experienced periodontist, blinded to treatment allocation. Prior to patient enrolment, the examiner performed a calibration exercise on training cases with duplicate measurements at identical sites to verify repeatability before study measurements commenced. During the study, a standardized probing technique and the same periodontal probe were used for all measurements.

Intraoperative clinical assessments included the depth of the bone defects, as well as the number of remaining bony walls.

### 2.3. Surgical Procedure

A minimally invasive approach incorporating papilla-preservation principles was used. The flap design was selected according to interdental space width: a simplified papilla preservation flap was adopted in narrow embrasures, while a modified papilla preservation flap was used in wider spaces. After papillary incision, full-thickness flaps were carefully elevated to expose only the coronal portion of the residual bony walls, minimizing tissue trauma and preserving soft-tissue integrity to promote wound stability and favorable healing. Thorough defect debridement and root surface instrumentation were performed with a combination of ultrasonic and hand instruments. After debridement, the root surface in both groups was conditioned with 24% EDTA gel (PrefGel^®^, pH 6.7; Straumann, Basel, Switzerland) for 2 min and thoroughly rinsed with sterile saline. In Group 1, hyaluronic acid (hyaDENT BG^®^, BioScience GmbH, Germany) was applied to the exposed root surface, and the xenogeneic bone substitute (Bio-Oss Collagen^®^, Geistlich) was pre-mixed with hyaDENT BG^®^ and placed into the intrabony defect. In Group 2, enamel matrix derivative (Emdogain^®^, Straumann) was applied from the base of the defect over the exposed root surface, and Bio-Oss Collagen^®^ pre-mixed with Emdogain^®^ was placed to fill the defect. In both groups, the flap was repositioned and secured to achieve primary closure.

Procedures were carried out by two surgical teams, each led by an experienced periodontist. To mitigate operator bias, each team treated an equal number of defects in both treatment groups. Although surgeons and patients were not blinded, perioperative steps were standardized across teams via a shared protocol covering flap design, debridement/root surface management, biomaterial placement, suturing, and postoperative care.

### 2.4. Radiographic Measurements

The diagnosis of periodontitis was radiographically confirmed. Standardized periapical radiographs were obtained before surgery and at the 6-month follow-up using the long-cone paralleling technique with a positioning device, maintaining the same X-ray unit, sensor, and exposure settings to ensure reproducibility. Measurements were performed in DBSWIN, version 5.10.1, by one calibrated examiner blinded to treatment allocation. The defect depth was defined as the linear distance from the base of the defect to the level of the interproximal bone crest of the adjacent tooth, and the defect angle as the angle formed by the root surface and a line along the corresponding bony wall. Bone fill (BF) was assessed as the difference between the postoperative and baseline bone levels, each measured relative to the cemento-enamel junction (CEJ). Examiner calibration was completed prior to patient enrolment using duplicate measurements on training cases to verify repeatability.

### 2.5. Statistical Analysis

Data were analyzed using IBM SPSS Statistics (Version 22). The significance level was set at *p* < 0.05 for hypothesis testing, indicating that null hypotheses would be rejected at this threshold. The following statistical methods were employed:

- Descriptive Analysis: This analysis included estimates of central tendency (such as mean, median) and dispersion (including standard deviation, range) to provide a comprehensive overview of the data characteristics.
- Distribution Normality Assessment: The Shapiro–Wilk non-parametric test was applied to evaluate the distribution type of the data. Given the small sample size, a normal distribution was not expected.
- Comparative Analysis for Independent Samples: The Mann–Whitney non-parametric test was applied to test for statistically significant differences between the two treatment groups. This test was specifically chosen due to the non-normal distribution of the data, as identified by the Shapiro–Wilk test.
- Comparative Analysis for Related Samples: The Wilcoxon signed-rank test was used to comparison of clinical parameters within the groups of patients with the same therapy, comparing their status prior to intervention and following the treatment. This test was specifically chosen due to the non-normal distribution of the data, as identified by the Shapiro–Wilk test.

The study flow is presented in Supplementary Figure S1 according to the CONSORT format (adapted for a non-randomized clinical design). A completed CONSORT 2025 checklist is provided in the Supplementary Materials. The study followed the CONSORT 2025 reporting recommendations [32].

## 3. Results

**Figure 1:**
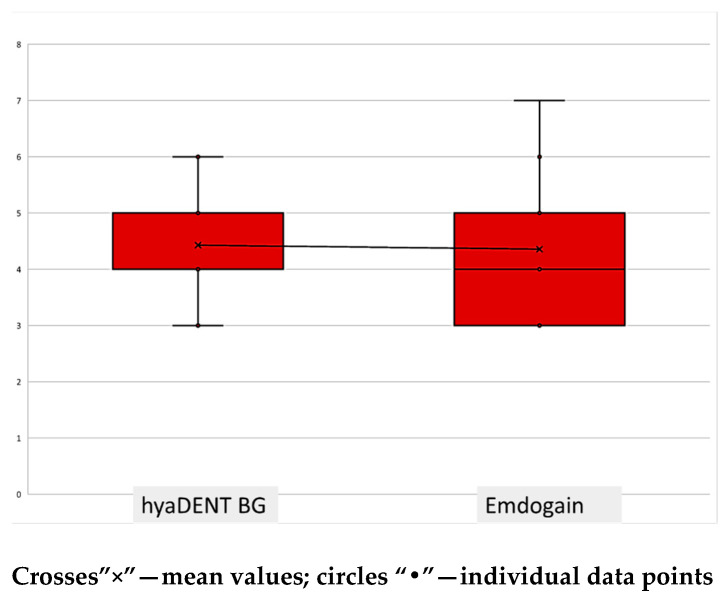
Intraoperative measurements of defect depth for both study groups.

Seventeen patients diagnosed with Stage III periodontitis (grades B or C), aged between 30 and 61 years, were enrolled in the study. In total, 28 intrabony defects were treated (HyaDent BG: 14 defects; Emdogain: 14 defects). Assignment to the treatment groups was non-random, based on case availability (convenience sample). Analyses were performed at the site level. Seven patients contributed sites only to the HyaDent BG group, nine only to the Emdogain group, and one contributed sites to both; the latter patient’s sites were excluded from between-group comparisons to avoid within-patient dependence. Because some patients contributed more than one defect, the age of each patient was recorded for each treated defect. The mean age was 47.15 years in the HyaDent BG group and 40.92 years in the Emdogain group. Since the age distribution in the HyaDent BG group was not normal, a non-parametric test was applied, showing that the difference between groups was not statistically significant (*p* = 0.101). At baseline, there were no statistically significant between-group differences in clinical or radiographic parameters, nor in the intraoperative measurements. The mean PPD at study entry was 7.2 mm in both groups (*p* = 0.92), and baseline CAL averaged 7.7 mm and 7.2 mm, respectively, in the hyaDENT BG group and in the Emdogain group (*p* = 0.362). Other parameters, including gingival recession and the radiographic measure (R1), showed no between-group differences (all *p* ≥ 0.26; Table 1). Defects were comparable in intraoperative depth and in baseline radiographic defect angle (Table 2), consistent with the overlapping distributions shown in Figure 1.

Defect morphology was also similar between groups. In the hyaDENT BG group, six two-wall and eight three-wall intrabony defects were managed; in the Emdogain group, eight two-wall and six three-wall defects. These distributions reinforce the comparability at baseline and intraoperatively, providing a sound basis for evaluating treatment effects.

At 6 months postoperatively, all treated sites underwent standardized re-evaluation. Within each treatment group, paired pre- to postoperative changes in probing depth, clinical attachment level, and radiographic bone fill were quantified at the defect level. Both interventions—hyaDENT BG–assisted Bio-Oss Collagen and Emdogain-assisted Bio-Oss Collagen— showed significant within-group improvements (Table 3 and Table 4).

In the hyaDENT BG group, probing depth decreased from 7.29 to 3.07 mm (mean reduction 4.22 mm), clinical attachment gain averaged 4.36 mm, and the radiographic defect measure declined from 5.00 to 0.57 mm (a reduction of 4.43 mm). In the Emdogain group, probing depth decreased from 7.43 to 2.50 mm (mean reduction 4.93 mm), clinical attachment gain averaged 4.57 mm, and the radiographic measure reached 0.14 mm (a reduction of 4.57 mm). Taken together, these paired changes indicate pronounced pocket reduction, attachment gain, and radiographic defect fill within each group.

Between-group comparisons at the 6-month follow-up showed no statistically significant differences in clinical or radiographic outcomes (Table 5). Mean PPD was 2.92 mm in the hyaDENT BG group versus 2.54 mm with Emdogain (*p* = 0.234; absolute difference 0.38 mm). Mean CAL was 3.00 mm versus 2.85 mm (*p* = 0.762; difference 0.15 mm). Gingival recession was identical in both groups (0.31 mm).

The radiographic residual defect (R2) measured 0.38 mm versus 0.15 mm (*p* = 0.479; difference 0.23 mm), and radiographic bone fill was 4.69 mm versus 4.54 mm (*p* = 0.650; difference 0.15 mm).

**Figure 2:**
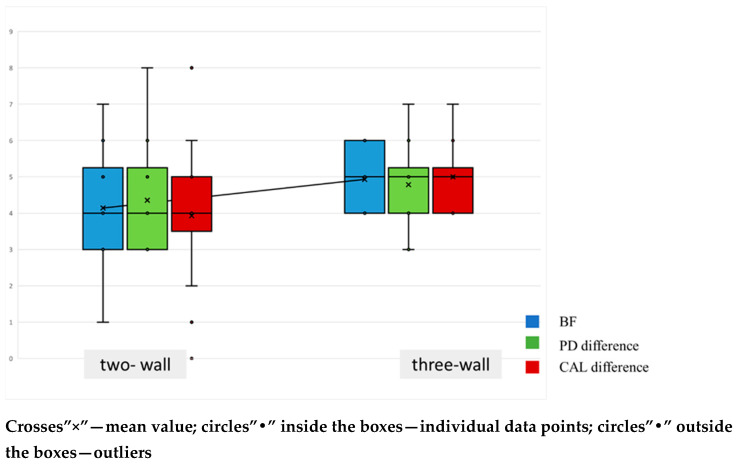
Changes in Bone Fill (BF), Probing Pocket Depth (PD difference), and Clinical Attachment Level (CAL difference) based on the number of bony walls.

Subsequently, analyses were stratified by defect type to determine whether two- or three-wall morphology affected the outcomes of the surgical procedure. Defect morphology was evenly represented: 14 two-wall and 14 three-wall intrabony defects were treated. Six-month change scores were analyzed by morphology for radiographic bone fill, probing depth, and clinical attachment level. As depicted in Figure 2, all three parameters improved in both morphologies: radiographic bone fill increased, PD decreased, and CAL gain was achieved. The distributions for two- and three-wall defects substantially overlap for all three outcomes, indicating broadly comparable improvements. Mean markers suggest only a modest tendency toward greater change in three-wall defects; however, this pattern should be interpreted cautiously, as no between-morphology differences reached statistical significance.

Outcomes at 6 months were stratified by defect angle using a 35° cutoff (Table 6). Postoperative values did not differ significantly between ≤35° and >35° defects for PPD2, CAL2, radiographic bone fill, or residual defect.

**Figure 3:**
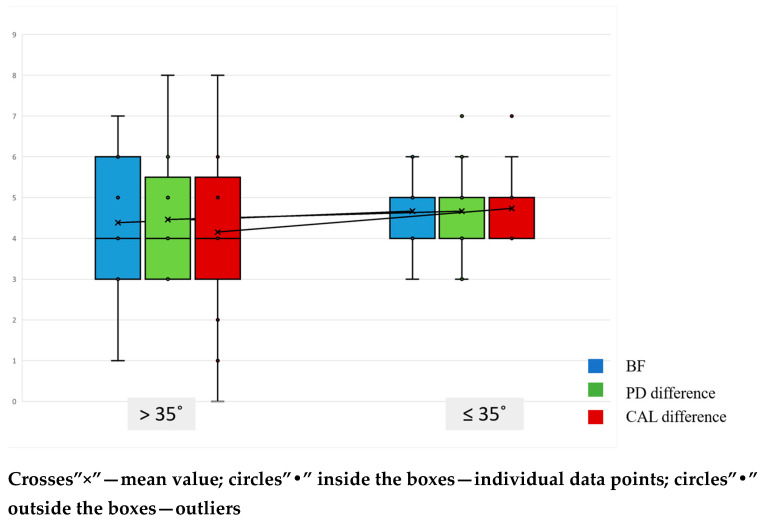
Changes in Bone Fill (BF), Probing Pocket Depth (PD difference), and Clinical Attachment Level (CAL difference) based on defect angle.

The box-and-whisker plots show substantial overlap between angle strata, consistent with these results (Figure 3). Nevertheless, wider defects (>35°) displayed a non-significant tendency toward higher residual PD and CAL and lower radiographic bone fill compared with narrower defects (≤35°).

## 4. Discussion

The current study evaluated clinical and radiographic outcomes after periodontal surgery for angular intrabony defects using either enamel matrix derivative or hyaluronic acid gel, each combined with a xenogeneic bone substitute. Over the 6-month follow-up, both treatment groups exhibited significant within-group improvements (all *p* < 0.001). However, between-group comparisons showed no statistically significant differences in postoperative values or mean changes (all *p* > 0.24). Overall, these findings suggest comparable clinical and radiographic outcomes for the two treatment protocols.

The use of enamel matrix derivative and hyaluronic acid in periodontal surgery has been widely reported in recent years [10,20,24,25,33].

One of the most widely cited clinical comparisons between hyaluronic acid dental gel and Emdogain^®^ was conducted by Pilloni et al. [33]. In this randomized controlled clinical trial, 32 intrabony defects were treated and followed for 24 months, providing a valuable long-term perspective on treatment efficacy. The results demonstrated a statistically significant improvement in clinical attachment levels (*p* < 0.001) for both therapeutic modalities. However, the control group—treated with Emdogain^®^—exhibited a more substantial reduction in periodontal pocket depth (*p* = 0.001), indicating slightly superior performance in that specific parameter.

Another recent randomized clinical trial directly comparing the regenerative outcomes of cross-linked hyaluronic acid and Emdogain^®^ was conducted by Rodríguez-A et al. [34]. This study is particularly relevant as it included a large sample of 53 intrabony defects and featured an extended follow-up period of 18 months. Both treatment modalities resulted in statistically significant improvements in probing pocket depth, clinical attachment level, and radiographic bone fill (*p* < 0.001). Although slightly greater early CAL gains were observed in the Emdogain^®^ group, the hyaluronic acid group demonstrated comparable long-term outcomes, suggesting that HA may serve as an effective alternative to EMD in the regenerative management of intrabony periodontal defects.

In the present study, both regenerative approaches led to clinically meaningful improvements in periodontal parameters after six months, consistent with the findings of Pilloni et al. and Rodríguez-A et al. Despite the shorter observation period in our study (6 months vs. 18–24 months), the patterns of clinical improvement were similar to those previously reported, although the limited sample size warrants cautious interpretation.

Whereas the above-mentioned trials assessed the regenerative potential of the two biomolecules as stand-alone adjuncts to open-flap debridement, the present investigation differs in its combined regenerative approach. In our study, both biomaterials were used together with a xenogeneic bone substitute (Bio-Oss Collagen^®^) to enhance space maintenance and support bone ingrowth. The combined use of a bioactive agent with a collagen-containing xenogeneic scaffold aligns with the concept of scaffold-mediated regeneration, whereby the matrix provides space maintenance and a template for cellular infiltration and bone ingrowth. Preclinical evidence indicates that collagen filling can modulate alveolar bone remodeling and favor new bone formation, reinforcing the biological rationale for the present protocol [35]. Moreover, histological data demonstrate that growth factors interact favorably with collagen carriers, supporting coordinated soft- and hard-tissue healing and providing a mechanistic rationale for pairing biologically active agents with collagen-based scaffolds [36]. Collectively, these findings support the concept that bioactive molecules combined with a collagenous scaffold may exert complementary effects—the scaffold stabilizing the defect and guiding bone ingrowth, and the bioactive agent modulating cellular events that underpin periodontal regeneration.

The absence of statistically significant differences between the two treatment groups may reflect a convergence of biological mechanisms. Both cross-linked hyaluronic acid and enamel matrix derivatives modulate cell adhesion, migration, and proliferation within the wound environment, promoting fibroblast/osteoblast activity and stabilizing the blood clot [37,38]. Moreover, a recent systematic review and meta-analysis reported that adjunctive hyaluronic acid, compared with open-flap debridement alone, yields greater clinical attachment gain and greater probing-depth reduction, with effects most evident at 6 months [39]. In further support of these outcomes, a randomized controlled trial evaluated the adjunctive use of 0.8% hyaluronic acid gel in periodontal surgery following initial non-surgical therapy and re-evaluation. Defects were randomly assigned to receive a modified Widman flap with either HA gel (test) or placebo (control). The study demonstrated a significant improvement in clinical attachment level (*p* < 0.05) in favor of the HA-treated sites, although no statistically significant differences were observed for probing depth or bleeding on probing (*p* > 0.05). Importantly, the authors emphasized clinical attachment gain as a key early treatment outcome and used cone-beam computed tomography (CBCT) at 6–12 months to confirm radiographic bone fill and structural regeneration [40].

Overall, the available evidence supports the biological plausibility of the comparable clinical outcomes observed in this preliminary study, particularly when both bioactive agents are combined with a collagen-based scaffold that may attenuate differences in regenerative potential. From a clinical perspective, HA may offer advantages in cases where the use of EMD is limited by cost, availability, or patient preference. EMD is sensitive to contamination with blood, which may reduce its effectiveness if hemostasis is incomplete during application [41]. In contrast, HA is biocompatible with blood and maintains its viscosity and adhesion in moist conditions, providing greater handling flexibility and clinical predictability [42]. Moreover, the synthetic, non-animal-derived composition of HA eliminates concerns related to religious or ethical restrictions associated with the porcine origin of EMD.

However, these observations should be interpreted with caution. The study’s non-randomized, convenience-based design introduces a risk of selection bias, as participants were assigned to treatment groups based on case availability rather than random allocation. The relatively small sample size, determined pragmatically by the availability of eligible patients and research funding, further limits statistical power and increases the possibility of a Type II error, where true between-group differences may have gone undetected.

Although all clinical measurements were obtained by a single calibrated examiner to minimize variability, repeat manual probing with a UNC-15 periodontal probe may still be susceptible to small measurement errors due to variations in angulation, insertion force, or tissue inflammation.

Additionally, the follow-up period of 6 months provides useful short-term insight into healing dynamics but does not allow conclusions regarding long-term stability of regenerative outcomes.

Finally, while the exclusion of patients with systemic diseases, heavy smoking, or poor oral hygiene strengthened internal validity by reducing confounding factors, it also limits external validity. Therefore, these results should be considered preliminary and mainly applicable to medically stable patients with good oral hygiene undergoing regenerative therapy under controlled conditions. Looking ahead, emerging evidence highlights the biological potential of several adjuncts—hyaluronic acid, enamel matrix proteins, growth factors, bone substitutes, platelet lysates, and selected pharmacologic agents [5,7,8,9,11,12,19,21,30]. Moreover, reports indicate that combining such biomolecules can promote favorable healing, enhance clinical attachment, facilitate bone formation, and deliver additional benefits in contemporary periodontal therapy [5,9,18,23,24,34,38,39,40,43]. Future research should aim to confirm these findings through larger, randomized controlled clinical trials with longer follow-up periods and standardized protocols. In addition, histological and biomolecular analyses would help clarify the underlying regenerative mechanisms and the true extent of new bone and cementum formation achieved with each biomaterial combination.

## 5. Conclusions

The literature contains limited data on the comparative evaluation of hyaDENT BG and Emdogain^®^, particularly when used in combination with bone substitute materials as adjuncts in regenerative periodontal surgery. Within the limitations of the present non-randomized study and its small sample size, both treatment protocols yielded statistically significant clinical and radiographic improvements at 6 months. These preliminary findings suggest that hyaluronic acid gel may serve as a potential alternative to Emdogain^®^ in regenerative periodontal therapy. However, these findings should be interpreted with caution, and confirmation through larger, randomized, and long-term clinical trials is required to establish definitive conclusions regarding comparative efficacy and long-term stability.

## Figures and Tables

**Table 1:** Pretreatment baseline data.

Parameter (mm)	hyaDENT BG	Emdogain	*p*-Value
	Average (*n* = 13)	Average (*n* = 13)	
PPD1	7.23 (95%CI [6.62; 7.84])	7.23 (95%CI [6.48; 7.98])	0.920
CAL1	7.69 (95%CI [7.18; 8.21])	7.15 (95%CI [5.90; 8.41])	0.362
GR1	0.46 (95%CI [0.15; 0.78])	0.38 (95%CI [0.00; 0.91])	0.418
R1	5.08 (95%CI [4.56; 5.60])	4.62 (95%CI [3.67; 5.56])	0.264

Abbreviations: PPD1—periodontal pocket depth before; CAL1—clinical attachment loss—before; GR1—gingival recession—before; R1—radiographic analysis—before; *n*—number of bone defects.

**Table 2:** Defect characteristics.

Parameter	hyaDENT BG	Emdogain	*p*-Value
	Average (*n* = 13)	Average (*n* = 13)	
Operative depth (mm)	4.54 (95%CI [4.01; 5.07])	4.31 (95%CI [3.55; 5.06])	0.479
Defect angle (°)	36.00 (95%CI [32.53; 39.47])	37.15 (95%CI [33.28; 41.03])	0.801

Abbreviations: *n*—number of bone defects.

**Table 3:** Outcomes for group 1.

hyaDENT BG Parameter (mm)	Before	After	*p*-Value
	Average (*n* = 14)	Average (*n* = 14)	
**PPD**	7.29 (95% CI [6.71; 7.86])	3.07 (95% CI [2.59; 3.55])	0.001 **
**CAL**	7.50 (95% CI [6.87; 8.13])	3.14 (95% CI [2.70; 3.59])	0.001 **
**R**	5.00 (95% CI [4.49; 5.51])	0.57 (95% CI [0.03; 1.11])	0.001 **

Abbreviations: PPD—periodontal pocket depth; CAL—clinical attachment loss; R—radiographic analysis; *n*—number of bone defects; ** statistically significant difference: *p* < 0.01.

**Table 4:** Outcomes for group 2.

Emdogain Parameter (mm)	Before	After	*p*-Value
	Average (*n* = 14)	Average (*n* = 14)	
**PPD**	7.43 (95% CI [6.62; 8.24])	2.50 (95% CI [2.01; 2.99])	0.001 **
**CAL**	7.36 (95% CI [6.12; 8.59])	2.79 (95% CI [2.22; 23.35])	0.001 **
**R**	4.71 (95% CI [3.82; 5.60])	0.14 (95% CI [0.00; 0.35])	0.001 **

Abbreviations: PPD—periodontal pocket depth; CAL—clinical attachment loss; R—radiographic analysis; *n*—number of bone defects; ** statistically significant difference: *p* < 0.01.

**Table 5:** Clinical and radiographic outcomes at postoperative follow-up.

Parameter (mm)	hyaDENT BG	Emdogain	*p*-Value
	Average (*n* = 13)	Average (*n* = 13)	
**PPD2**	2.92 (95% CI [2.54; 3.31])	2.54 (95% CI [2.01; 3.07])	0.243
**CAL2**	3.00 (95% CI [2.65; 3.35])	2.85 (95% CI [2.25; 3.44])	0.762
**GR2**	0.31 (95% CI [0.02; 0.60])	0.31 (95% CI [0.02; 0.60])	-
**R2**	0.38 (95% CI [0.00; 0.78])	0.15 (95% CI [0.00; 0.38])	0.479
**BF**	4.69 (95% CI [4.24; 5.15])	4.54 (95% CI [3.70; 5.38])	0.650

Abbreviations: PPD2—periodontal pocket depth—after; CAL2—clinical attachment loss—after; GR2—gingival recession—after, R2—radiographic analysis—after, BF—bone filling; *n*—number of bone defects.

**Table 6:** Differences in Clinical and Radiographic Parameters According to Defect Angle.

Parameter	≤35°	>35°	*p*-Value
	Average (*n* = 14)	Average (*n* = 14)	
**PPD2 (mm)**	2.53 (95% CI [2.07; 3.00])	3.08 (95% CI [2.56; 3.60])	0.142
**CAL2 (mm)**	2.80 (95% CI [2.28; 3.32])	3.15 (95% CI [2.67; 3.64])	0.413
**BF (mm)**	4.67 (95% CI [4.17; 5.16])	4.38 (95% CI [3.38; 5.39])	0.683
**R2 (mm)**	0.27 (95% CI [0.00; 0.60])	0.46 (95% CI [0.00; 0.99])	0.650

Abbreviations: PPD2—periodontal pocket depth—after; CAL2—clinical attachment loss—after; BF—bone filling; R2—radiographic analysis—after; *n*—number of bone defects

## Data Availability

The original contributions presented in the study are included in the article, further inquiries can be directed to the corresponding author.

## Supplementary Materials

The following supporting information can be downloaded at: https://www.mdpi.com/article/10.3390/jfb16120431/s1, Figure S1: CONSORT 2025 Flow Diagram; Table S1. Distribution of treated sites by jaw, tooth type, and treatment group.

## Abbreviations

The following abbreviations are used in this manuscript:

EMD	enamel matrix derivatives
PGA	propylene glycol alginate
HA	hyaluronic acid
PPD	probing pocket depth
BoP	bleeding on probing
FMPS	Full Mouth Plaque Score
FMBS	Full Mouth Bleeding Score
CAL	clinical attachment loss
F	furcation involvement
M	tooth mobility
GR	gingival recession
R	radiographic analysis
BF	bone filling
CBCT	cone- beam computed tomography
OFD	open- flap debridement
