# Supplementary material for: Comparative Evaluation of Hyaluronic Acid (hyaDENT BG® Gel) and Enamel Matrix Proteins (Emdogain®) in the Regenerative Treatment of Angular Bone Defects Using Xenograft (Bio-Oss Collagen®)—A Clinical Trial
Source: J Funct Biomater. 2025 Nov 24;16(12):431. doi: 10.3390/jfb16120431 (PMC12734394; doi:10.3390/jfb16120431)
Supplement: Supplementary file 1 [file jfb-16-00431-s001.zip › CONSORT_2025_flow_diagram-updated.pdf]

Figure S1: CONSORT 2025 Flow Diagram

This was a **non-randomized**, controlled clinical study. Participants were assigned to treatment groups based on case availability and clinical feasibility rather than random allocation.

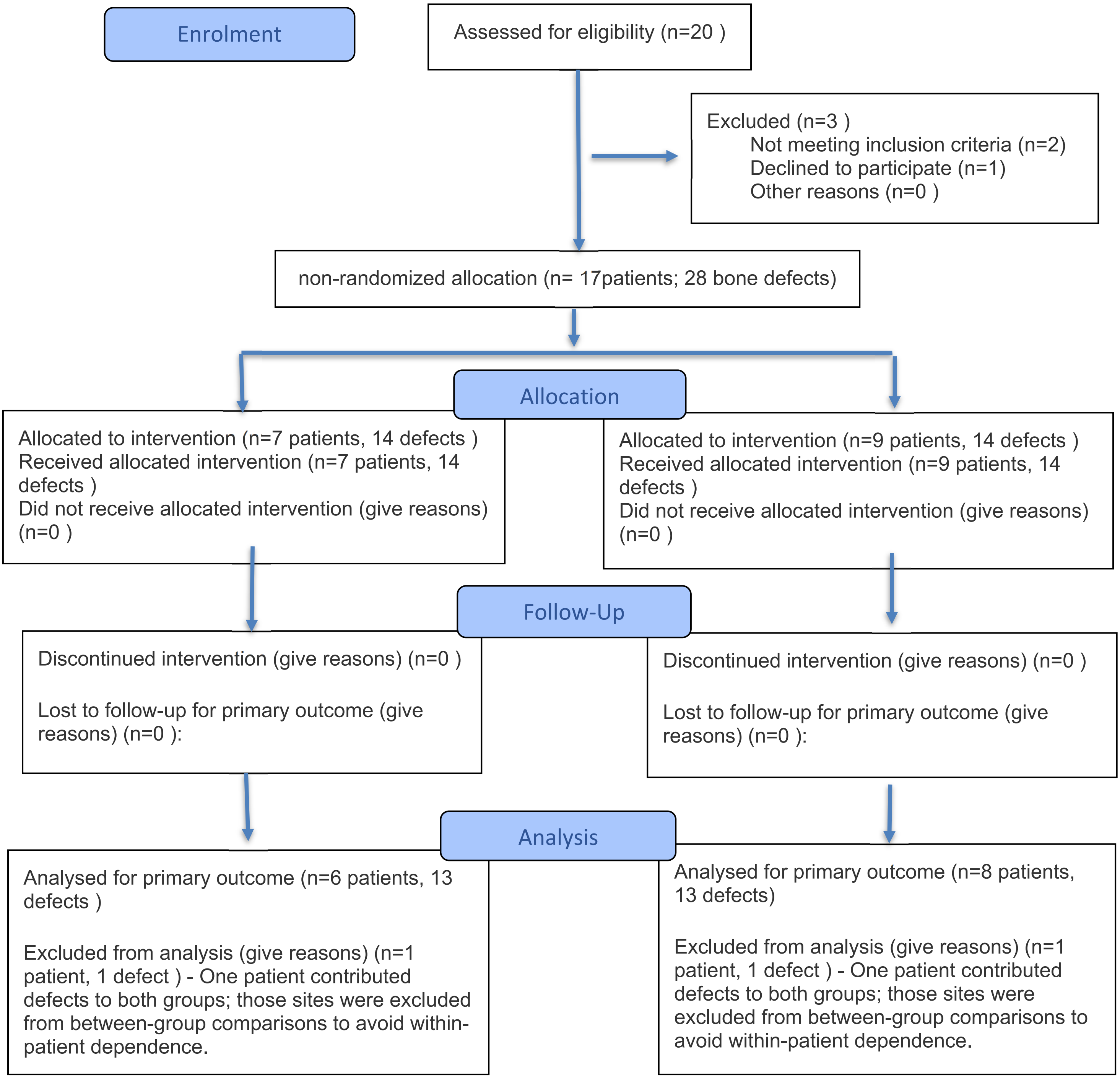

Citation: Hopewell S, Chan AW, Collins GS, Hróbjartsson A, Moher D, Schulz KF, et al. CONSORT 2025 Statement: updated guideline for reporting randomised trials. BMJ. 2025; 388:e081123. https://dx.doi.org/10.1136/bmj-2024-081123
© 2025 Hopewell et al. This is an Open Access article distributed under the terms of the Creative Commons Attribution License (https://creativecommons.org/licenses/by/4.0/), which permits unrestricted use, distribution, and reproduction in any medium, provided the original work is properly cited.
