# Supplementary material for: Comparative Evaluation of Hyaluronic Acid (hyaDENT BG® Gel) and Enamel Matrix Proteins (Emdogain®) in the Regenerative Treatment of Angular Bone Defects Using Xenograft (Bio-Oss Collagen®)—A Clinical Trial
Source: J Funct Biomater. 2025 Nov 24;16(12):431. doi: 10.3390/jfb16120431 (PMC12734394; doi:10.3390/jfb16120431)
Supplement: Supplementary file 1 [file jfb-16-00431-s001.zip › jfb-3933492-supplementary.pdf]

Table S1. Distribution of treated sites by jaw, tooth type, and treatment group

JAW / TOOTH TYPE	GROUP 1: BIO-OSS + HYADENT BG	GROUP 2: BIO-OSS + EMDOGAIN	TOTAL
**MAXILLA – INCISORS**	4	0	**4**
**MAXILLA – CANINES**	0	1	**1**
**MAXILLA – PREMOLARS**	3	2	**5**
**MAXILLA – MOLARS**	1	3	**4**
**MANDIBLE – INCISORS**	2	1	**3**
**MANDIBLE – CANINES**	0	0	**0**
**MANDIBLE – PREMOLARS**	1	1	**2**
**MANDIBLE – MOLARS**	3	6	**9**
**TOTAL SITES PER GROUP**	**14**	**14**	**28**
